# Genome-Wide Profiling Reveals Alternative Polyadenylation of Innate Immune-Related mRNA in Patients With COVID-19

**DOI:** 10.3389/fimmu.2021.756288

**Published:** 2021-10-27

**Authors:** Sanqi An, Yueqi Li, Yao Lin, Jiemei Chu, Jinming Su, Qiuli Chen, Hailong Wang, Peijiang Pan, Ruili Zheng, Jingyi Li, Junjun Jiang, Li Ye, Hao Liang

**Affiliations:** ^1^ Biosafety Level-3 Laboratory, Life Sciences Institute & Guangxi Collaborative Innovation Center for Biomedicine, Guangxi Medical University, Nanning, China; ^2^ Guangxi Key Laboratory of AIDS Prevention and Treatment, School of Public Health, Guangxi Medical University, Nanning, China

**Keywords:** COVID-19, alternative polyadenylation, immunity, alternative splicing, APA regulator

## Abstract

The coronavirus disease 2019 (COVID-19) pandemic has caused many deaths worldwide. To date, the mechanism of viral immune escape remains unclear, which is a great obstacle to developing effective clinical treatment. RNA processing mechanisms, including alternative polyadenylation (APA) and alternative splicing (AS), are crucial in the regulation of most human genes in many types of infectious diseases. Because the role of APA and AS in response to severe acute respiratory syndrome coronavirus 2 (SARS-CoV-2) infection remains unknown, we performed *de novo* identification of dynamic APA sites using a public dataset of human peripheral blood mononuclear cell (PBMC) RNA-Seq data in COVID-19 patients. We found that genes with APA were enriched in innate immunity -related gene ontology categories such as neutrophil activation, regulation of the MAPK cascade and cytokine production, response to interferon-gamma and the innate immune response. We also reported genome-wide AS events and enriched viral transcription-related categories upon SARS-CoV-2 infection. Interestingly, we found that APA events may give better predictions than AS in COVID-19 patients, suggesting that APA could act as a potential therapeutic target and novel biomarker in those patients. Our study is the first to annotate genes with APA and AS in COVID-19 patients and highlights the roles of APA variation in SARS-CoV-2 infection.

## Introduction

SARS-CoV-2 causes the respiratory disease known as COVID-19. By the start of August 2021, there had been more than 200 million cases and at least 4,200,000 deaths caused by COVID-19 around the world.

mRNA-processing events, including APA and AS, play key roles in various diseases (1, 2). APA is a widespread mechanism of gene regulation that generates different 3’ ends of transcripts (3). APA leads to the production of distinct protein isoforms and repressing gene expression (4, 5). APA regulators control APA by binding to the APA site during mRNA processing (6). AS enables an mRNA to differentiate into isoforms that may have different biological functions (3). Therefore, APA and AS are involved in transcriptional gene regulation. Overall, the diversity of the transcriptome and proteome is enhanced by APA and AS, which are two important regulatory mechanisms for many biological processes. Indeed, through APA, a single gene can encode multiple different 3’ ends of transcripts. Polyadenylation affects numerous aspects of mRNA metabolism, including transcription termination by RNAP II, mRNA stability, and the efficiency of translation (7). APA upregulates target genes through miRNA repression escape (6). Shortening of the average 3′UTR length and widespread APA in response to virus infection have been reported (8). The expression level of genes with APA is altered and enriched in immune-related categories including interferon (8). Interferons have shown *in vitro* and *in vivo* antiviral effect against SARS-CoV-2, and they have been suggested as a potential treatment for COVID-19 patients (9, 10). Moreover, APA has an effect on viral replication and plays a crucial role in the antiviral innate immune response (8). The mechanisms of changes in APA are considered to be regulated in *cis* through genetic aberrations (11) and in *trans* by regulatory proteins in response to dynamic environmental changes (12). To date, there has been no research on whether APA plays an essential role in patients infected with SARS-CoV-2.

Similar to alternative polyadenylation, hundreds of host genes showed AS upon viral infection (13). For instance, infection of human cells with influenza A virus induced a broad program of alternative splicing of host genes (14). However, the in-depth molecular basis of infection and pathogenesis of SARS-CoV-2 in human cells has not been further explained. It has also been reported that SARS-CoV-2 disrupts mRNA splicing *in vitro (* 1). There is an urgent need to determine whether these AS anomalies correlate with clinical features.

Based on the above information, we aimed to reveal overall dynamic changes in APA and AS in COVID-19 patients. DaPars (6) and rMATS (15) were used to directly detect APA and AS events from RNA-Seq data. Our findings will contribute to our understanding of the pathogenesis, potential molecular targets, and development of new APA-based therapeutic targets of COVID-19.

## Materials and Methods

### Data Extraction

The sequencing data used in this study were retrieved from two large-scale multiomic studies of COVID-19 (16, 17), in which 128 patients admitted to Albany Medical Center in Albany, NY were collected for moderate to severe respiratory issues presumably related to SARS-CoV-2 infection from April 6, 2020 to May 1, 2020. Patients who were positive (n = 102) and negative (n = 26) for the virus were assigned to the COVID-19 and non-COVID-19 groups, respectively. Due to missing sequencing data for 2 positive patients, a total of 126 cases were included in this study. RNA-Seq data of the COVID-19 cohort mentioned above were available at the SRA database SRP279280 (https://www.ncbi.nlm.nih.gov/sra/) (16). To validate our results, independent RNA-Seq data for PBMCs from the non-COVID-19 and COVID-19 groups were downloaded from the GSA database CRA002390 (https://bigd.big.ac.cn/gsa/) (17). The reads were mapped to the hg38 human genome using HISTA2 (v2.1.0) (18). We used StringTie (v1.3.4d) to calculate the TPMs (transcripts per million) of Ensembl annotated genes (19). Differential gene expression analyses were performed using DESeq2 according to the read counts of each gene determined by HTSeq (18). Genes with FDR (false discovery rate) ≤ 0.05 and mean CPM (Couts per Million) > 100 were determined to be differentially expressed genes, as we descripted previously (20) (**Table S1**).

### Alternative Polyadenylation Analysis

We used APA to link genetic variation to variations in gene expression and disease risk. DaPars is a bioinformatic algorithm dedicated to *de novo* identification and quantification of dynamic APA events using standard RNA-Seq (6). In this study, we used DaPars (0.9.1) to infer, identify and quantify APA in RNA-Seq data for COVID-19 patients and non-COVID-19 cohorts. The APA with FDR ≤ 0.05 and Δ|PDUI|>0.1 were determined as the significantly different APA between COVID-19 and non-COVID-19 as described previously (21). The percentage of distal polyA site usage index (PDUI) was defined as follows:


$$PDUI=\frac{L}{L+S}$$

S and L are the abundances of transcripts with proximal distal polyA sites in each sample. The following is a detailed description of the options used with DaPars. FDR_cutoff=0.05, PDUI_cutoff=0.1 (**Table S2**).

### Differential Alternative Splicing Analysis

The main types of AS include exon skipping (SE), intron retention (IR), alternative 5’ splice site (A5SS), alternative 3’ splice site (A3SS) and mutually exclusive exon (MXE). In total, 126 samples, including 100 COVID-19 and 26 non-COVID-19 patient samples, were used for analysis. We used hisat2 (v2.1.0) for sequence alignment and rMATS for AS analysis (15). Using FDR ≤ 0.05 as the threshold, the AS analysis results were screened to obtain a matrix of differential AS genes and expression levels. We ran rMATS on bam as input data. The following is a description of the options used with rMATS: FDR ≤ 0.05, Read length = 51, and Thread=32 (**Table S3**). The data were visualized using the Integrative Genomics Viewer (IGV) tool (22).

### GO Function Enrichment and KEGG Pathway Analyses

To describe potential different mechanisms between those with and without COVID-19, differential APA genes, AS genes, anddifferentially expressed genes screened in the previous step were used for GO function enrichment analysis and KEGG pathway enrichment analysis using The Database for Annotation, Visualization and Integrated Discovery (DAVID, https://david.ncifcrf.gov/) and Metascape (https://metascape.org/gp/index.html#/main/step1) (23, 24). The Benjamini-Hochberg (BH) method was used to obtain the FDR value, and FDR ≤ 0.05 was taken as the threshold. Pathway/GO terms satisfying this condition were defined as those significantly enriched among differentially expressed genes. The top hits with the most significant enrichment (the lowest p-value) are shown in a histogram.

### Principal Component Analysis

Principal component analysis (PCA) is used to transform original correlating variables into two uncorrelated principal components by linear transformation and to characterize the contribution rate by variance (also called the eigenvalue); we selected the first two principal components for analysis according to the contribution rate. After standardizing the input alternative splicing PSI (percent spliced in index) and PDUI values, the R packages factominer (V2.4) and factoextra (v1.0.7) were applied to reduce the PCA dimension of AS and APA genes, and the first and second principal components were extracted for two-dimensional visualization.

### Analyses of the Relationship of APA With DEG

We performed cumulative distribution analysis of log2 fold changes in mRNA expression in R as we described previously (20). The empirical cumulative distribution functions of the log2-fold mRNA expression values were computed using the ecdf function, and the corresponding p‐value was included in the plot. miRNA-binding sites were predicted by TargetScanHuman (25), and Hypergeometric Optimization of Motif EnRichment (HOMER) software was used for motif enrichment analysis (26).

## Results

### Dynamic APA Events in COVID-19 Patients

**Figure 1:**
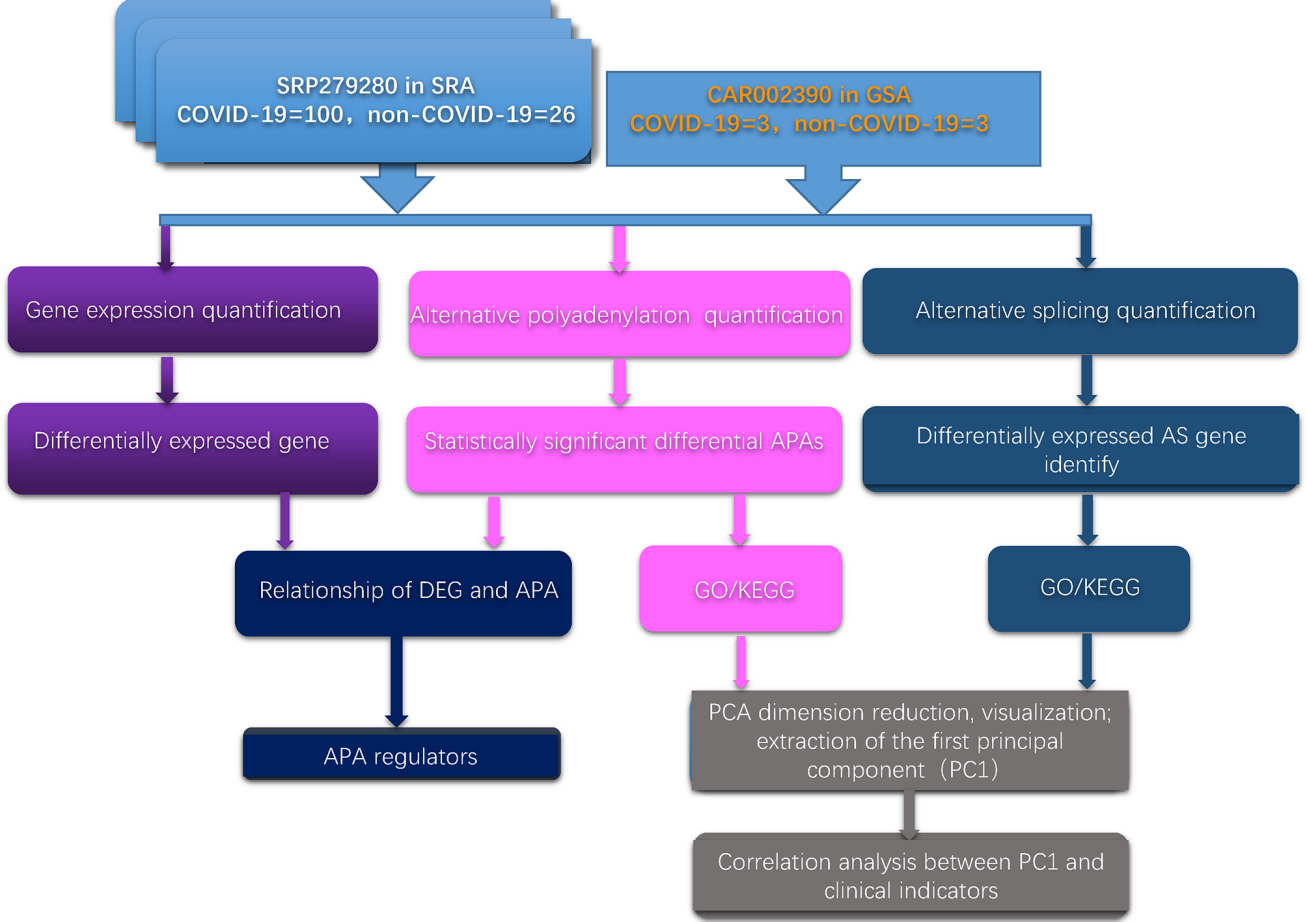
Schematic flow chart demonstrating the computational framework.

First, we developed a computational framework to systematically analyze the dynamic changes in APA, gene expression and AS in two independent databases. The computational framework is illustrated in **Figure 1**. The detailed method is given in the *Materials and Methods* section.

**Figure 2:**
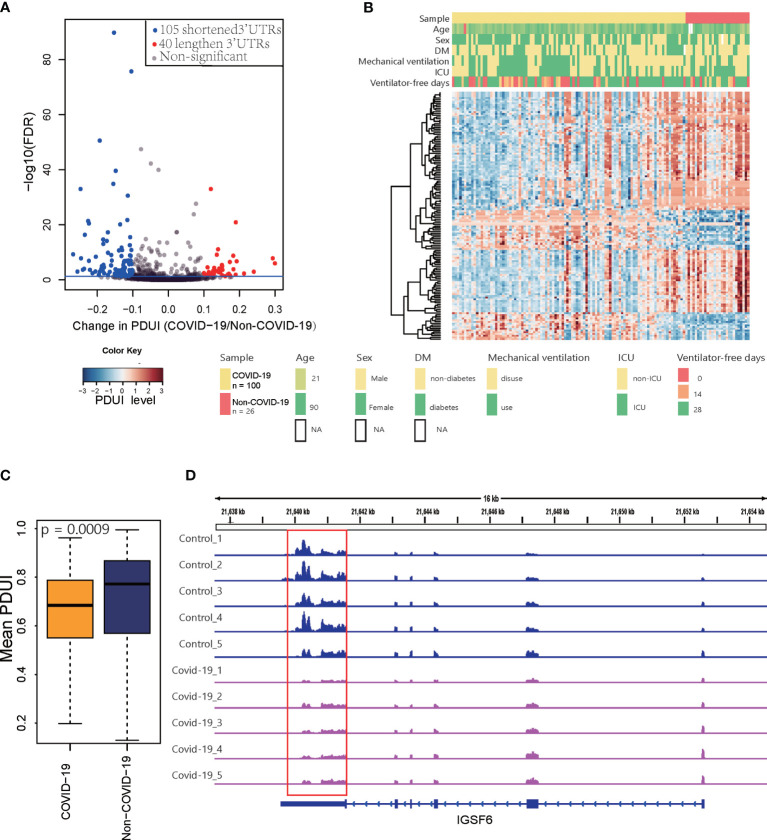
Differential APA analysis for COVID-19. **(A)** Volcano plot showing the log2FC of PDUI and the statistical significance of ΔPDUI between COVID-19 patients and non-COVID-19 patients. Red indicates upregulated APA sites; blue indicates downregulated APA sites. **(B)** A heat map showing the APA index of all the genes that displayed significant APA. **(C)** Average 3’UTR length of non-COVID-19 and COVID-19 patients. **(D)** Tracks for IGSF6 in 5 COVID-19 samples and 5 control samples randomly selected from 128 samples.

We performed *de novo* identification and quantification of dynamic APA events using existing RNA-Seq data for PBMCs from COVID-19 patients. Based on data for 100 COVID-19 patients and 26 non-COVID-19 subjects collected in GEO, we detected 145 sites that were significant dynamic APA events (FDR ≤ 0.05 and Δ|PDUI|>0.1) in COVID-19 (**Figures 2A, B**) (**Table S2**). We quantified the degree of difference of APA in COVID-19 as a change in the percentage of distal polyA site usage index (ΔPDUI), which could identify shortening (negative index) or lengthening (positive index) of 3′UTRs. As shown in **Figures 2A, B**, in COVID-19 samples, most dynamic APA events presented a shorter 3′UTR. The mean PDUI value was significantly lower in the COVID-19 group than in the non-COVID-19 group(**Figure 2C**). Additionally, COVID-19 patients with shorter 3′UTRs had a decreasing number of ventilator-free days compared with those with longer 3′UTRs. COVID-19 patients with shorter 3′UTRs also had higher intensive care unit (ICU) admission rates. We concluded that APA is related to the clinical characteristics of COVID-19. Furthermore, the canonical polyA signal AATAAA was successfully identified by HOMER motif enrichment analysis of dynamic APA sites (**Figure S1A**) (27), AATAAA was found to be the most predominant motifs in human APA events in pervious study (8). One example of a changed APA event in COVID-19 compared with non-COVID-19 is given for the IGSF6 gene (Immunoglobulin Superfamily Member 6) (**Figure 2D**), with a shorter 3′UTR predominating in COVID-19 samples compared with matched non-COVID-19 samples in GEO (16). Furthermore, we validated this result with another independent data in GSA (17) (**Figure S1B**). Collectively, these analyses revealed global changes in APA in COVID-19 patients.

### Functional Analysis of APA in COVID-19

**Figure 3:**
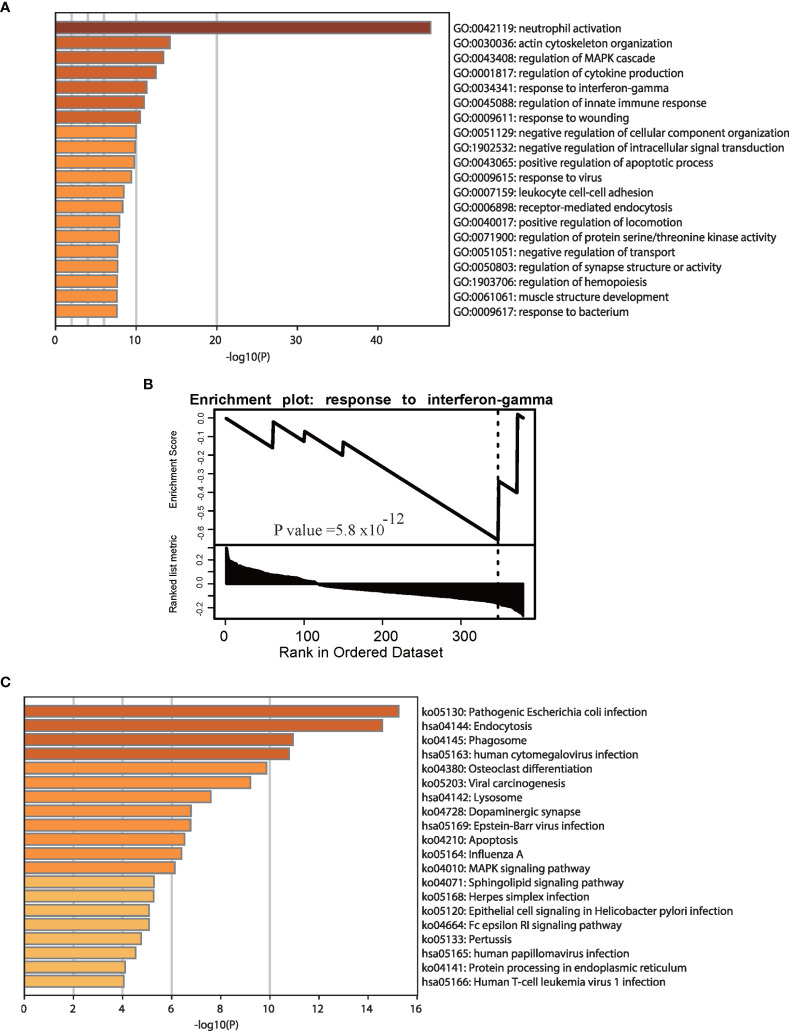
Enrichment analyses of genes with significant APA changes. GO **(A)** and GSEA **(B)** enrichment analyses of genes with significant APA changes. **(C)** KEGG pathway enrichment analyses of genes with significant APA changes. The GO and pathway terms are displayed on the x-axis and are significantly enriched at −log10 (p value).

According to previous studies, the type I interferon (IFN) response characterizes the local immune response phase due to SARS-CoV-2 attack (28). The IFN system caused by TNF-a and IFN-g mirrors the tissue damage and inflammation that occur in COVID-19 samples (29), and most GO and pathway enrichment analyses based on gene expression data have revealed that interferon terms are enriched in COVID-19 patients (17). We performed GO and pathway enrichment analyses to explore changes based on APA in response to SARS-CoV-2 infection and found that APA is associated with key biological processes and pathways of COVID-19 (**Figure S2**). **Figure 3A** shows significant enrichment of significantly different APA in biological processes associated with innate immune responses, such as neutrophil activation, regulation of the MAPK cascade and cytokine production, response to IFN-gamma and the innate immune response. In fact, viruses infecting vertebrate hosts must overcome the IFN-mediated antiviral response before replicating and propagating to new hosts (30). As shown in **Figure 3B**, the GSEA results showed that genes with APA were significantly enriched in response to IFN-gamma. Interestingly, this result is similar to previous GO results based on differentially expressed genes in COVID-19 (17). To further investigate the specific innate immune regulatory APA in COVID-19, we selected representative samples to track the IFN-related genes CD14, IL6 and IFNGR1(**Figure S3**). In addition, we performed KEGG pathway enrichment analysis using genes with significantly different APA (**Figure 3C**). This analysis indicated that genes expressing APA were significantly enriched in pathogenic *Escherichia coli* infection, endocytosis, phagosome human cytomegalovirus infection, Epstein-Barr virus infection and human T-cell leukemia virus 1 infection. We concluded that APA was involved in the IFN signaling pathway in the antiviral innate immune response of COVID-19 patients.

### APA and DEGs in COVID-19

**Figure 4:**
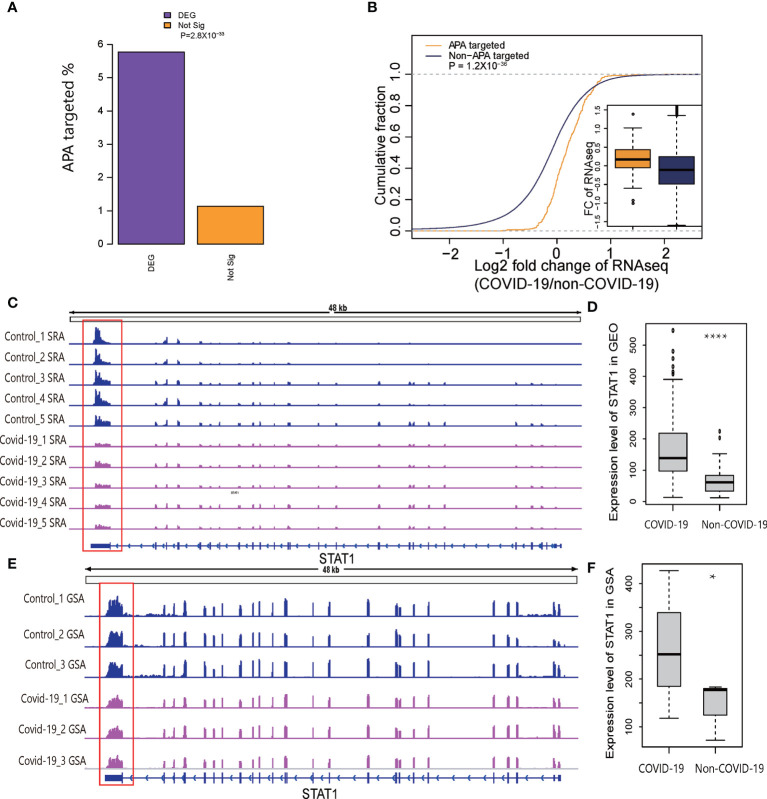
Relationship of APA and DEGs in COVID-19. **(A)** Barplot representing the percentages of the DEGs or nonsignificant genes enriched for significant APA sites. **(B)** Plot of the cumulative fraction of log2-fold change of gene expression ratios in COVID-19 patients comparing genes with significant APA sites *versus* those overlapping without significant APA sites. The p value of a two-tailed Wilcoxon test is indicated. **(C)** Tracks displaying the read coverage for the STAT1 gene in samples randomly selected from SRP279280. **(D)** Expression level of STAT1 in COVID-19 and non-COVID-19 patients in GEO. **(E)** Tracks displaying the read coverage of validation RNA-Seq data (CRA002390) for the STAT1 gene. **(F)** Expression level of STAT1 in COVID-19 and non-COVID-19 patients in GSA. *P < 0.05, ****P < 0.05*10^-4.

It is widely accepted that 3′UTR shortening through APA may upregulate target genes *via* miRNA repression escape (6). Because the results of immune-related pathway enrichment for APA were similar to the results of pathway enrichment for the differentially expressed genes, we speculated that APA affected the expression level of immune-related genes in COVID-19 patients. To address this possibility, we examined differentially (DEGs) and nondifferentially expressed genes to assess whether the APA sites were overrepresented in COVID-19 associated DEGs. As expected, almost 6% of significantly DEGs underwent APA, whereas only 1% of nondifferentially expressed genes underwent APA, indicating a significantly enriched occurrence of APA target sites among DEGs (*p*=2.8×10^−33^, two-tailed chi-square test; **Figure 4A**), suggesting that APA affects host gene expression level. Moreover, the fold change ratios of the genes with APA were significantly upregulated compared to genes without APA target sites in COVID-19 (*p*= 1.2×10^−36^, two-tailed Wilcoxon test; **Figure 4B**). We also observed that 77% of genes with shorter 3′UTRs in COVID-19 samples lost at least 1 predicted miRNA-binding site, indicating that APA might upregulate parental genes by escaping miRNA repression (**Figure S4A**).

It has been reported that the STAT1/STAT3 axis is required for TNF-α- and IFN-γ-induced inflammatory cell death-PANoptosis in COVID-19 (29). Thus, we manually compared APA in multiple IFN-related genes, especially STAT1 in two independent datasets to verify our results (**Figures 4C, E**). We found a shorter 3′UTR for STAT1 and significantly higher gene expression levels in two independent datasets (**Figures 4D, F**). Since there were too many samples, we randomly selected five representative samples to show the trends. The findings are consistent with the theoretical hypothesis that 3′UTR shortening through APA may upregulate target genes through miRNA repression escape. Collectively, these analyses revealed that APA play an important role in the regulation of gene expression in COVID-19 patients.

### Regulatory Factors of APA

The core polyadenylation trans factors include the activity of 3’ processing factors. The 3’ processing complex consists of over 20 core proteins (27), including cleavage and polyadenylation specificity factor (CPSF), cleavage factors (CFim and CFIIm), Two cytoplasmic poly(A)-binding proteins (PABPC1 and PABPC4), cleavage stimulatory factor or cleavage stimulation factor (CSTF), poly(A)-binding protein nuclear 1 (PABPNl), FIP1L1, PCF11 and SYMP. Previous studies have found that alteration in the expression of some 3’ processing factors caused significant changes in poly (A) site choice (31).

**Figure 5:**
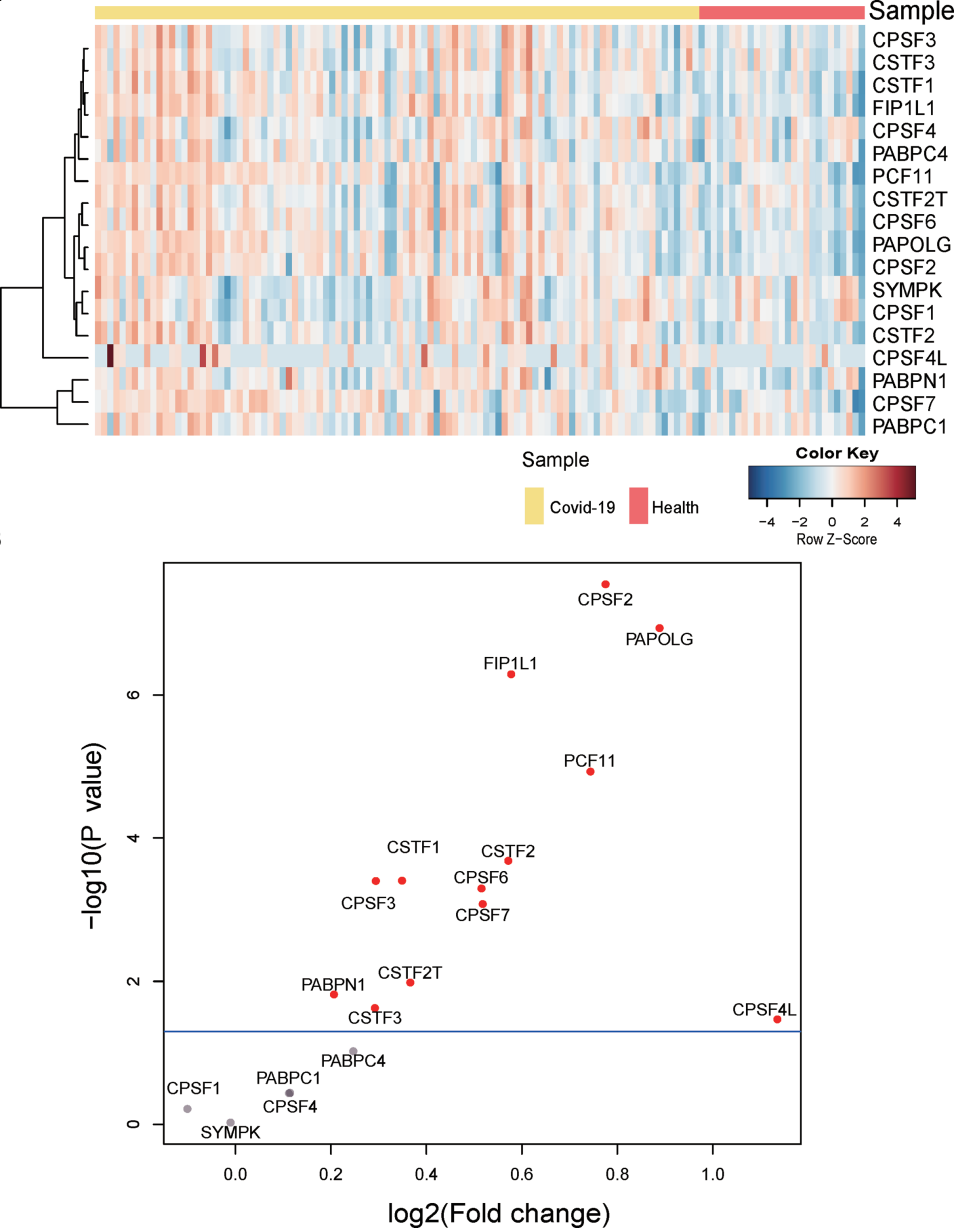
Gene expression of APA regulators. **(A)** Heatmap of gene expression of regulators of APA. **(B)** Volcano plot of differentially expressed APA regulators. Significant regulators (p value ≤ 0.05) are highlighted in red.

To investigate whether APA changes with the expression level of 3’ processing factors in COVID-19 patients, we analyzed changes in expression of 3’ processing factors in PBMCs from COVID-19 patients. **Figures 5A, B** show that the expression level of most 3’ processing factors was significantly higher in COVID-19 patients (*P* < 0.05), especially CPSF2, PAPOLG, FIP1L1, and PCF11. To validate our results, we analyzed RNA-Seq data from other independent COVID-19 datasets and observed the same results (**Figure S4B**), suggesting change of APA is associated with expression level of 3’processing factors in COVID-19 patients.

In brief, we concluded that high expression level of 3’ processing factors might be one of the reasons underlying genome-wide APA when patients are infected with SARS-CoV-2. Our results were consistent with previous studies that 3′UTR shortening correlated positively with high expression of polyadenylation factors (32).

### Performance Evaluation of APA and AS

**Figure 6:**
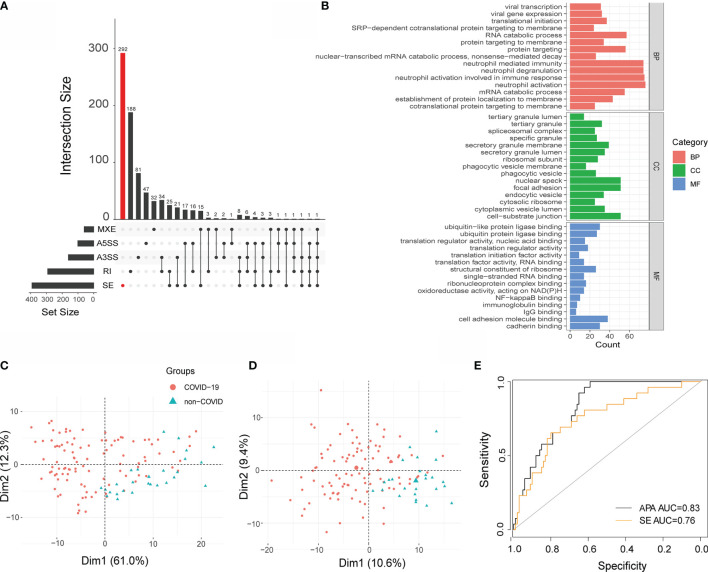
Performance of APA and SE analysis. **(A)** Bar plot showing the genes exhibiting AS during SARS-CoV-2 infection (at 5% FDR). **(B)** GO terms obtained from functional annotation analysis using R software. **(C)** Principal component analysis (PCA) of APA and SE **(D)**. Each dot represents one sample. Red: COVID-19, Green: non-COVID-19. **(E)** Receiver operating curve (ROC) plot of the performance based on accuracy using PC1 for APA and SE.

To further determine the power of APA in COVID-19 patients, we compared APA with AS, and AS has been proven to play an important role in COVID-19. We performed alternative splicing analysis for COVID-19 patients by using rMARTs (33), a computational tool employed to detect differential AS events based on RNA-Seq data. We detected a total of 806 events in the COVID-19 sample, including 640 unique AS events, which are 292 SE events, 188 RI events, 47 A5SS events, 81 A3SS events, and 32 MXE events (**Figure 6A**). Moreover, the pathways enriched in AS were immune related (**Figure 6B**) (**Table S3**). Considering that SE was found to be the most prevalent AS event, we conducted principal component analysis (PCA) to construct APA and SE signatures as described previously (34).

We identified two significant components that explained 61% and 12% of the APA variation. They also explained 10.6% and 9.4% of the SE variation, respectively. The first PC (principal component) mainly separated COVID-19 patients from non-COVID-19 patients with regard to APA and SE (**Figures 6C, D**). Then, principal component 1 was selected to act as a signature score predicting COVID-19 status. To further explore the diagnostic potential of the above dynamic APA and SE, we performed receiver operating characteristic (ROC) curve analysis. As illustrated in **Figure 6C**, the area under the curve (AUC) for discriminating COVID-19 from non-COVID-19 was 0.83 for APA, suggesting its high diagnostic potential in COVID-19. Furthermore, the AUC for SE was 0.76 (**Figure 6E**), which indicated that APA provided greater accuracy than SE in COVID-19. Furthermore, we investigated the association between the clinical outcomes of COVID-19 patients and APA conditions. We found that the APA index was significantly changed in the different hospital free days groups (**Figure S5A**). However, there were not significantly differences in the APA index between different ventilator free day groups and male *vs* female COVID-19 patients (**Figures S5B, C**). Hence, we fill the knowledge gap of regarding the role of APA in the clinical outcomes of COVID-19 patients.

## Discussion

In brief, we performed *de novo* identification of dynamic APA and AS using two independent datasets of PBMC RNA-Seq data from COVID-19 patients. We found that APA and gene expression of APA regulators in the hosts was perturbed in 100 COVID-19 patients. This study contributes to a better understanding of the activation and evasion of interferon responses by SARS-CoV-2, and this will be beneficial to the study of new treatment methods for COVID-19. Even though all of the in silico works were performed in two independent datasets, the limitations of our in silico work cannot be ignored. For instance, we could not demonstrate which APA regulator mainly regulates abnormal APA in COVID-19 patients. Wet experiments need to be conducted to demonstrate the detailed regulatory mechanism in future studies. At the same time, the effects of abnormal APA on phenotypes in human immune cell lines were also need to be conducted using wet experiments.

Transcriptome analysis of PBMCs can indicate numerous RNA mis-APA and mis-AS events, which may serve as disease-specific biomarkers (35). Although PBMCs are peripheral blood cells comprising T cells, B cells, NK cells, and monocytes, they are widely used to identify potential biomarkers for COVID-19 (36–38). For instance, RNA-Seq data for COVID-19 PBMCs indicated immune-related transcriptomic profiles (1). However, PBMCs contain many cell types, which is difficult to verify by wet experiments.

It was reported that antiviral pathways and interferon pathways play important roles in COVID-19 patients (38–41), but the exact mechanism remained unclear. Simultaneously, previous studies have found that APA and AS regulated multiple viral immune processes (7, 8, 13, 42–44). However, there is a lack of studies on whether APA affects SARS-CoV-2 infection. Innate lymphoid cells including neutrophils and macrophages secrete signaling factors that regulate innate and adaptive immune responses. Recent studies have reported that innate immunity may play a more central role in combating SARS-CoV-2 rather than adaptive immunity (45). Based on our results, APA may cause neutrophil activation by affecting the release of inflammatory factors from macrophages. APA may play a central role in innate immunity in COVID-19 patients. In addition, neutrophils can express costimulatory molecules and MHC-II after exposure to cytokines, such as IFN-γ (46). APA may also affect the release of antiviral factors such as interferon and exert antiviral effects. It has been reported that IFN-induced STAT1 phosphorylation remains intact in the presence of SARS-CoV-2 ORF6 (47) and that alteration of STAT1 increases susceptibility to virus infections because it is involved in various signaling pathways both upstream and downstream of IFN production. Interestingly, in COVID-19 patients, APA (and AS) genes were enriched in IFN related categories, especially pathogenic *Escherichia coli* infection, endocytosis, phagosome human cytomegalovirus infection, and Epstein-Barr virus infection signaling pathways.

We revealed that 3′UTR shortening through APA might play an essential role in COVID-19 and that APA upregulated target genes by facilitating miRNA repression escape in SARS-CoV-2 infection, which was consistent with previous report that 3′UTR shortening through APA might upregulate target genes by miRNA repression escape in other infectious diseases. Although previous studies showed that global 3′UTR shortening affects protein abundance (8), our study revealed that the impact of the 3′UTR on protein production may depend on the gene. However, APA of the genes confers different functions and needs further investigation. Most of the *trans* factors assessed were highly expressed in COVID-19 patients, in accordance with previous results that 3′UTR shortening was associated with highly expressed *trans* factors. For instance, the key poly-A trans-factor CSTF64 was significantly upregulated, leading to preferential 3′UTR shortening in tumors (6). The expression of 3′ processing factors was down-regulated when cells were infected by vesicular stomatitis virus, which might be one of the reasons underlying genome-wide APA when cells were infected with viruses (8). Meanwhile, we found that the expression level of 3′ processing factors was also altered in COVID-19 patients. Therefore, the question here is how SARS-CoV-2 disrupts APA and AS. According to the research conclusion that SARS-CoV-2 proteins could bind to transcription factor and splicing factors (1), we proposed the following hypothesis: SARS-CoV-2 proteins can bind to APA factors affecting the gene expression level of APA factors to regulate APA. Although SE has been reported to play a key role in SARS-CoV-2 infection (1), our PCA results of dynamic APA and SE showed that APA provided greater accuracy than SE (**Figure 6C**). This conclusion will contribute to a better understanding of mRNA-processing mechanisms in COVID-19 samples. Furthermore, APA may disrupt antigen presentation by MHC in infected cells, and interference with APA and AS might further aid SARS-CoV-2 in evading the host immune response. Our article contributes to the development of new APA-based therapeutic targets of COVID-19.

## Data Availability Statement

The original contributions presented in the study are included in the article/**Supplementary Material**. Further inquiries can be directed to the corresponding authors.

## Author Contributions

SA and YQL collected the omics data and conducted the analysis. YL and JC produced the diagrams. SA, LY, and JS wrote the draft of the manuscript. RZ, JL, QC, HW, PP, and HL revised the draft. JJ supervised the project. All authors contributed to the article and approved the submitted version.

## Funding

China Postdoctoral Science Foundation (2020M683623XB), The 64th Batch of the China Postdoctoral Science Foundation (2018M643382), Young Scientists Fund of the Guangxi Natural Science Foundation (2018GXNSFBA281014), Guangxi Bagui Scholar (to JJ), Guangxi Medical University Training Program for Distinguished Young Scholars (to JJ), Guangxi Science Fund for Distinguished Young Scholars (2018GXNSFFA281001), National Natural Science Foundation of China (82160389).

## Conflict of Interest

The authors declare that the research was conducted in the absence of any commercial or financial relationships that could be construed as a potential conflict of interest.

## Publisher’s Note

All claims expressed in this article are solely those of the authors and do not necessarily represent those of their affiliated organizations, or those of the publisher, the editors and the reviewers. Any product that may be evaluated in this article, or claim that may be made by its manufacturer, is not guaranteed or endorsed by the publisher.
