# Supplementary material for: Genome-Wide Profiling Reveals Alternative Polyadenylation of Innate Immune-Related mRNA in Patients With COVID-19
Source: Front Immunol. 2021 Oct 27;12:756288. doi: 10.3389/fimmu.2021.756288 (PMC8578971; doi:10.3389/fimmu.2021.756288)

Supplementary figure 1. (A) HOMER identifies the canonical polyA motif AATAAA with a significant p value (1x10-8) from APA sites predicted by DaPars. (B)Volcano plot showing different APAs between COVID-19 and Non-COVID-19 groups. Each dot indicates individual transcript, colored by red and blue when a transcript is significant lengthen or shortened 3'UTRs. Horizontal line represents FDR = 0.05 and vertical lines indicate of |ΔPDUI| = 0.1. (C) Bar plots showing enrichment p-values of 20 representative GO terms for genes with significantly different APA.

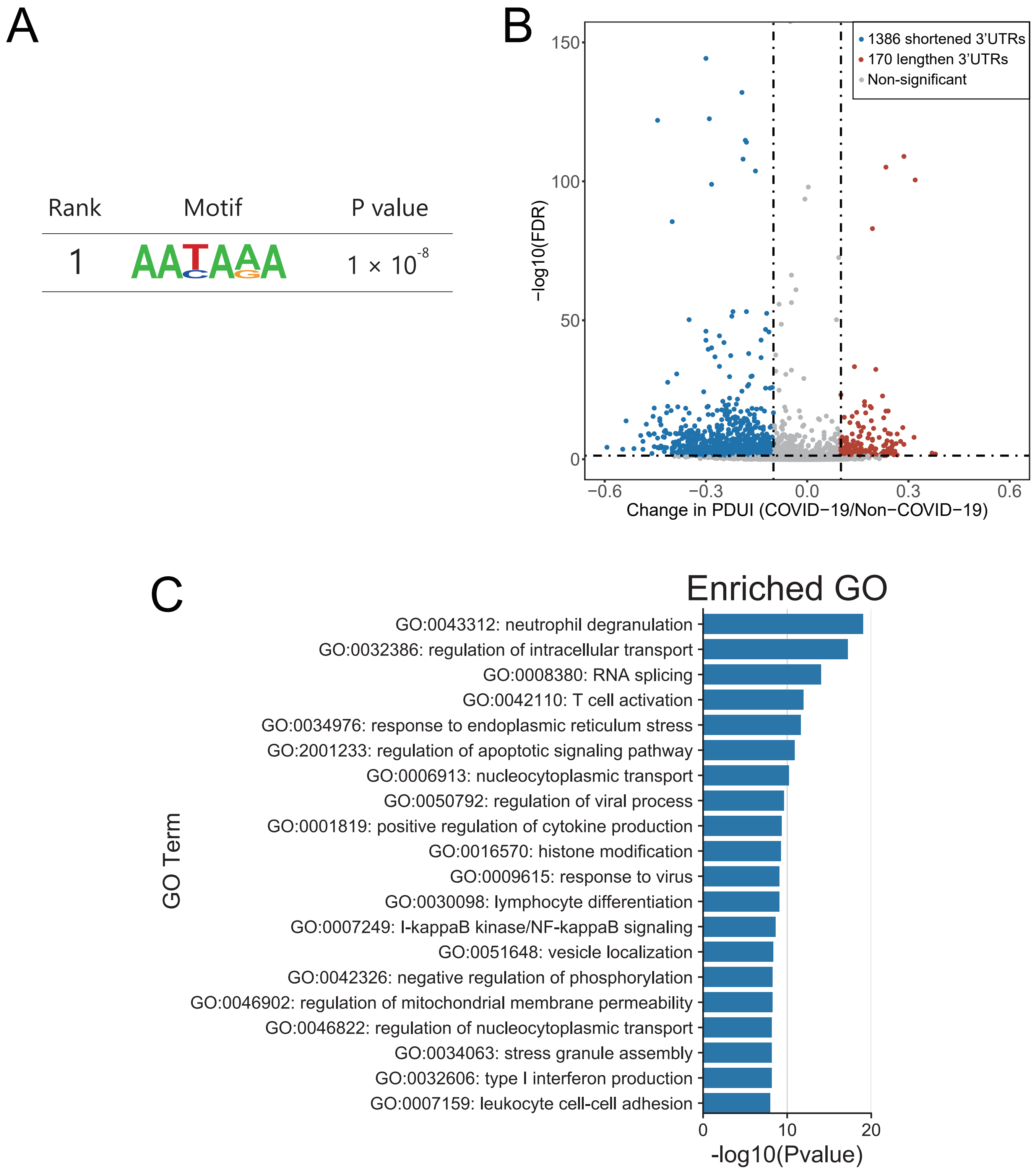

Supplementary figure 2. COVID-19-related biological pathway enrichment analysis of genes with significant APA changes using Metascape.

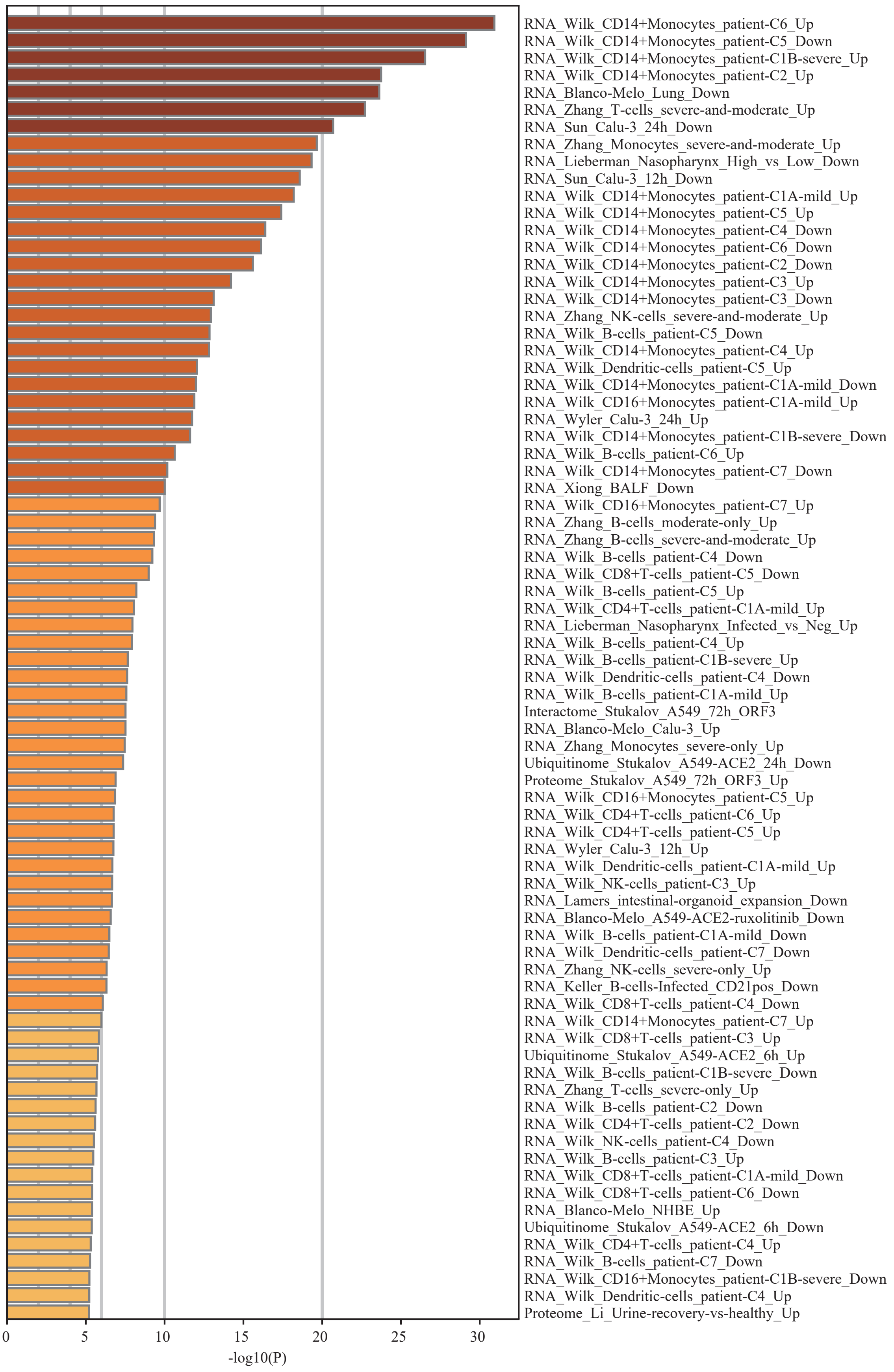

Supplementary figure 3. Tracks displaying the read coverage for the genes CD14, IL6 and IFNGR1 in samples randomly selected from SRP279280.

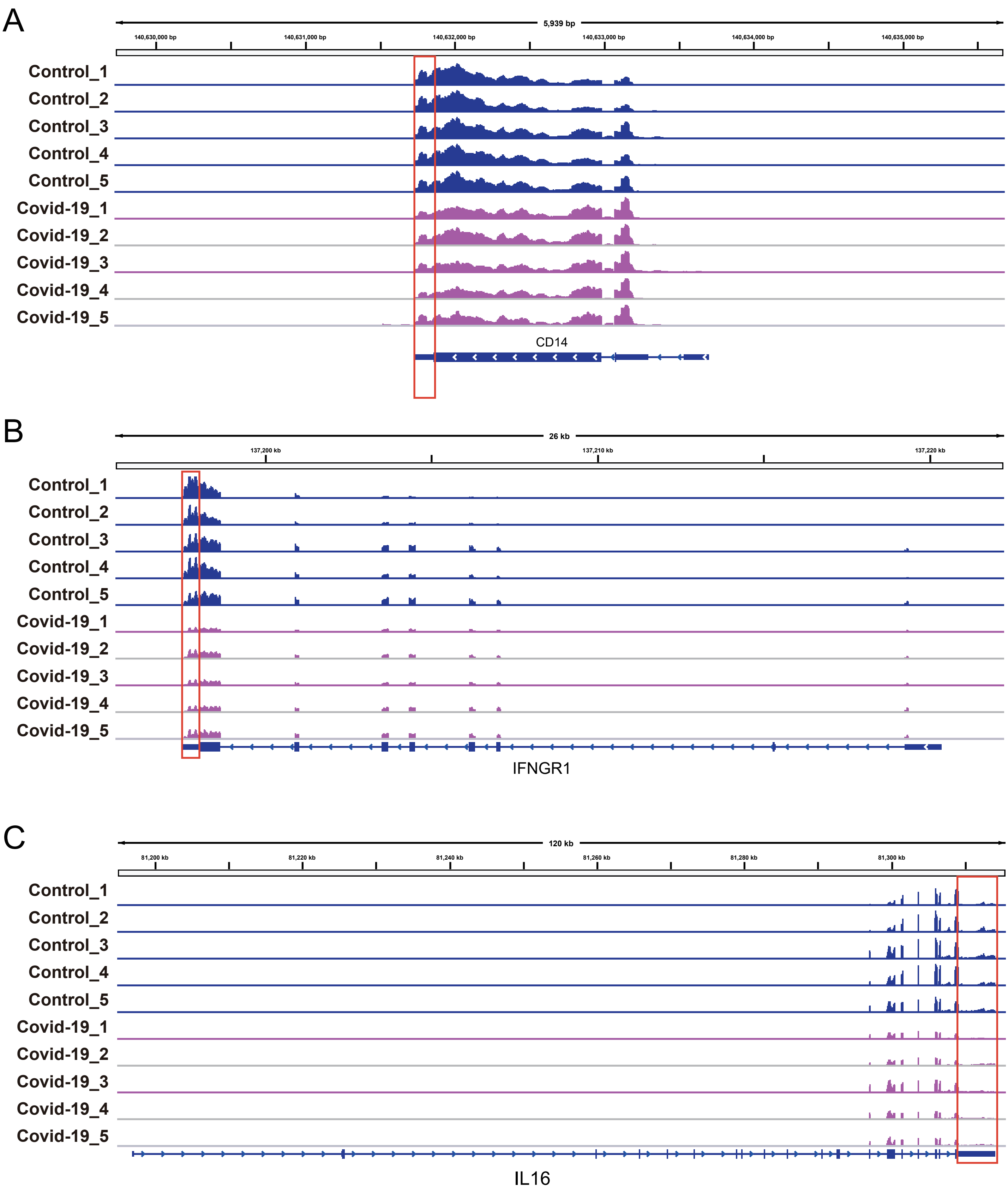

Supplementary figure 4. APA and corresponding miRNA-binding sites predicted by TargetScanHuman (A) and Volcano plot of differential APA regulator expression in GSA (B).

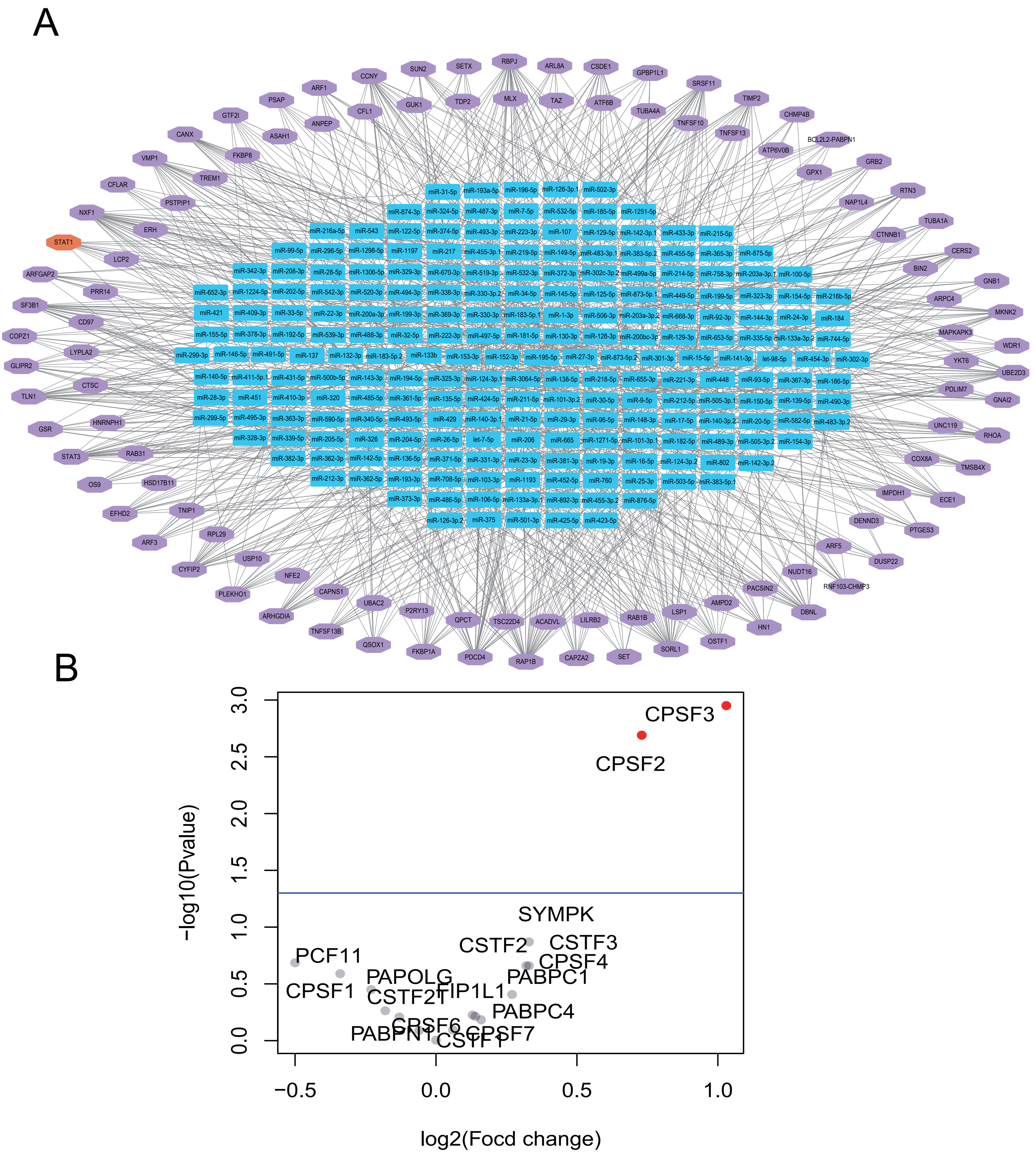

Supplementary figure 5. APA index and clinical outcomes of COVID-19 patients
(A, B) Boxplots showing the APA index of COVID-19 patients in different hospital free days and ventilator-free days. The results of the Wilcox signed rank sum test showed that the APA index was significantly different between the 10~20-day hospitalization group and the 30~40-day group (P=0.03). n.s means no significant difference. (C) Boxplot shows the comparison of APA index between male and female in COVID-19 and Non-COVID-19 groups. n.s means no significant difference

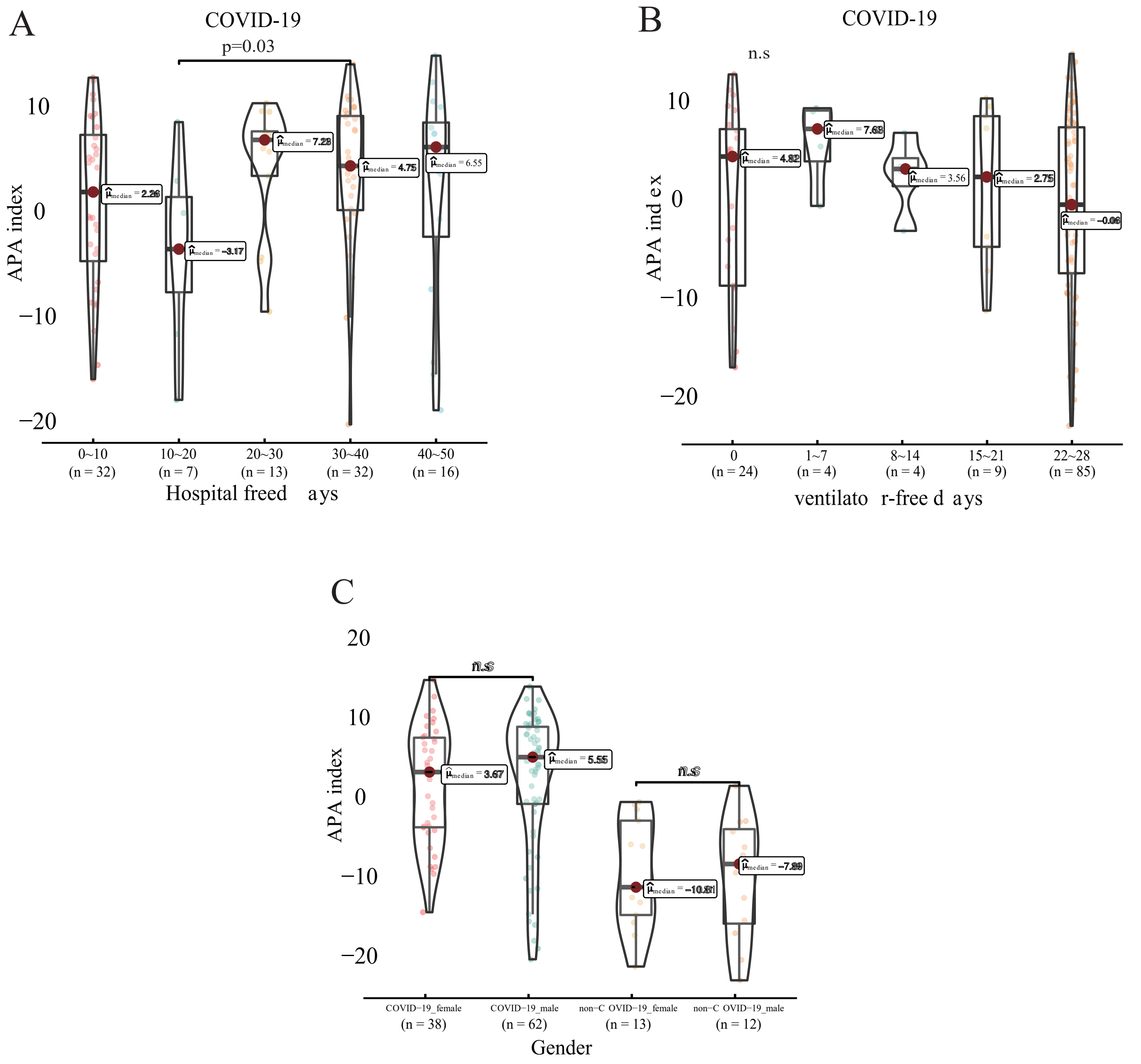

Supplement: Supplementary file 1 [file DataSheet_1.pdf]
